# Combining gene expression programming and genetic algorithm as a powerful hybrid modeling approach for pear rootstocks tissue culture media formulation

**DOI:** 10.1186/s13007-019-0520-y

**Published:** 2019-11-18

**Authors:** Saeid Jamshidi, Abbas Yadollahi, Mohammad Mehdi Arab, Mohammad Soltani, Maliheh Eftekhari, Hamed Sabzalipoor, Abdollatif Sheikhi, Jalal Shiri

**Affiliations:** 1 Department of Horticultural Science, Faculty of Agriculture, Tarbiat Modares University (TMU), Tehran, Iran; 2 Department of Horticultural Sciences, College of Aburaihan, University of Tehran (UT), Tehran, Iran; 3 Department of Irrigation and Drainage Engineering, College of Aburaihan, University of Tehran, Tehran, Iran; 4 Department of Nanobiotechnology, Faculty of Biological Sciences, Tarbiat Modares University (TMU), Tehran, Iran; 5 Department of Horticultural Sciences, College of Agriculture, Vali-e-Asr University of Rafsanjan, Kerman, Iran; 6 Department of Water Engineering, Faculty of Agriculture, University of Tabriz, Tabriz, Iran

**Keywords:** Gene expression programming, Radial basis function neural network, Genetic algorithm, Multiple linear regression, Pyrodwarf, OHF, Pear rootstock

## Abstract

**Background:**

Predicting impact of plant tissue culture media components on explant proliferation is important especially in commercial scale for optimizing efficient culture media. Previous studies have focused on predicting the impact of media components on explant growth via conventional multi-layer perceptron neural networks (MLPNN) and Multiple Linear Regression (MLR) methods. So, there is an opportunity to find more efficient algorithms such as Radial Basis Function Neural Network (RBFNN) and Gene Expression Programming (GEP). Here, a novel algorithm, i.e. GEP which has not been previously applied in plant tissue culture researches was compared to RBFNN and MLR for the first time. Pear rootstocks (Pyrodwarf and OHF) were used as case studies on predicting the effect of minerals and some hormones in the culture medium on proliferation indices.

**Results:**

Generally, RBFNN and GEP showed extremely higher performance accuracy than the MLR. Moreover, GEP models as the most accurate models were optimized using genetic algorithm (GA). The improvement was mainly due to the RBFNN and GEP strong estimation capability and their superior tolerance to experimental noises or improbability.

**Conclusions:**

GEP as the most robust and accurate prospecting procedure to achieve the highest proliferation quality and quantity has also the benefit of being easy to use.

## Background

Graft incompatibility and fruit quality are the present and future challenges that pear orchards are facing with them [1, 2]. In this framework, Pyrodwarf and OHF dwarfing rootstocks acquired from *Pyrus communis* were used. Pyrodwarf a hybrid of “Old Home” and “Gute Luise” is particularly tolerant to winter cold temperatures, calcareous soils and has a highly significant compatibility with all pear varieties. Moreover, it is characterized by a high precocity, good productivity, and producing uniform in size fruits with pear cultivars [3]. OHF as a cross between “Old Home” and “Farmingdale” cultivars, has reasonably resistance to fireblight, excellent anchorage and is compatible with all pear cultivars [4, 5]. Therefore, many problems with pear orchards are largely overcome by use of these rootstocks, and based on the above reasons, both rootstocks are recommended for high-density planting systems. Classic methods for propagation of pear such as cutting and layering which have been mostly used, are too expensive, time consuming and labor intensive [6]. Micropropagation is a reliable technique that plays a decisive role in rapid propagation of pear and researchers have claimed considerable success with this approach. The responses of pear cultivars to micropropagation are significantly variable, so that some cultivars have exhibited disorders like shoot tip necrosis [7], hyperhydricity [8–10], fascination [11] and hooked leaves [9], while some of them grow well [12].

According to the previous studies, [13] (MS) is a very common medium in pear micropropagation but it is not appropriate medium in all cases. That is why recent investigations have focused on optimization of the culture medium [10, 14, 15]. Several studies have been done on modifying MS medium by changing the level of plant growth regulators (PGRs) [8, 12, 16–18]. Wada et al. [19] found that increasing mesos nutrients content of MS medium improves multiplication rate in several pear cultivars and alleviates some physiological disorders. Reed et al. [7] adjusted MS medium by increasing mesos levels (1.5 × MS) and reducing nitrogen compounds (0.5–0.8 × MS) to produce shoots without physiological abnormalities in some pear genotypes. Wada et al. [20] found that some pear cultivars require high NO_3_^−^ (52 to 60 mM), low to moderate NH_4_^+^ (data not shown) and high mesos concentrations (1.5 × MS) for the best growth results. Another approach for optimization of MS medium was performed by [21] who suggested that adding meta-topolin (6–9 µM), an aromatic cytokinin, to MS medium increases significantly the multiplication rate and shoot quality in OHF-333 (another clonal selection of Old Home × Farmingdale).

Due to various nutrition necessities of different plant species, it is difficult to obtain an optimized culture medium. Therefore, it is important to employ a trustworthy modeling system to achieve the highest growth performance [15].

Biological systems are difficult to understand and model, largely due to the complexities of their system and non-linear nature; moreover, their dynamic characteristics are poorly understood. Usually, it is difficult to clarify biological interactions by traditional models and algorithms, particularly in the complex and noisy data set. Additionally, many studies have demonstrated that plant biology needs more attempts to find developing platforms for combining multidimensional data in order to draw biological interactions.

In recent decades, several meta-modeling techniques have been emerged as promising methods for modeling high dimensional and non-linear processes. Artificial neural networks (ANN) [22, 23], gene expression programming (GEP) [24–26], fuzzy logic (FL) [27] and statistical methodologies [20] are the best examples. Previous investigations have demonstrated that artificial intelligence (AI) based modeling approaches are vastly superior in modeling process to all other techniques [15, 28].

The capability to quickly and effectively emulate nonlinear trends in data operation have helped setting up AI technology as a reliable meta-modeling platform for surrogate modeling in a wide variety of practical assignment, including science and engineering, because of their ability to utilize learning algorithm and detect input–output relationships in intricate, nonlinear processes. Among the numerous aspects of the AI paradigms, ANN technique has a wide range of applications in the field of plant biology and prediction of bioprocess quantitative properties. Successful applications of this approach have been reported in many studies. Among others, ANN was found as a promising technique to assess the effects of mineral nutrients on plant growth and productivity [29]. ANN and Response surface method (RSM) were utilized to model and optimize fermentation media and the results showed that ANN performed significantly better than the RSM methodology [30]. ANN models superiority to traditional statistical analysis was confirmed for assessing the plant biological processes [31]. ANN was applied to model the effects of cultivar and exogenous auxin on in vitro rooting and acclimatization of some grapevine genotypes [32]. Culture media composition was modeled by using a hybrid ANN-genetic algorithm (ANN-GA) method and compared with regression techniques [10] and the superiority of the ANN-GA over the traditional methodology was proved. ANN was found as a very accurate method in modeling and predicting the composition of G × N15 rootstock proliferation culture medium [15].

Radial basis function neural network (RBFNN) is one of the most popular feed-forward ANN architectures in the literature for forecasting problems, among which the back-propagation (BP) algorithm is the most extensively used, and is a supervised learning model. The RBFNN can be considered as a special three-layer feed forward network which has certain advantages including better approximation capabilities, fast learning algorithms, simple network structures, and will not encounter the local minima problems [33, 34]. RBFNN can illuminate the complex law between the inputs and outputs through a fitting process that allows it to approximate any nonlinear function [35–38].

Although the reported approaches are fitting for modeling culture media, no approaches have been reported to provide effective and clear modeling results as well as explicit formulations of the studied phenomenon without presuming previous shape of the relationship [39, 40]. This induces us to use and suggest a novel method to bridge these gaps among contemporary paradigms. Genetic programming (GP) [41] is a fairly new soft computing approach for the behavior modeling of structural engineering problems. GP is an extension of GA which searches a program space instead of a data space. The main advantage of the GP-based approaches is their ability to generate prediction equations without assuming prior form of the relationship. Many researchers have employed GP and its variants to discover any complex relationships among experimental data [42–44]. Gene expression programming (GEP) [45] is a recent extension to GP. GEP evolves computer programs of different sizes and shapes encoded in linear chromosomes of fixed length. The GEP chromosomes are composed of multiple genes, each gene encoding a smaller subprogram [42]. The GEP approach is shown to be an efficient alternative to the traditional GP [45, 46].

The researches mentioned above exhibit the potential of AI methodologies as appropriate modeling tools for plant tissue culture. However, the literature survey revealed that no investigation has been done to use GEP for predicting new culture medium. This gave new impetus to the present study.

The purpose of this paper is to apply two soft computing techniques namely RBFNN and GEP and to compare their prediction accuracy to Multiple Linear Regression (MLR) method as well as using GA algorithm aiming to predict and optimize pear culture media. The main contributions of this paper are as follows:

- Investigating the application of the GEP and RBFNN for modeling the effects of macronutrients and hormones on in vitro culture of Pyrodwarf and OHF rootstocks.
- Development of GEP-GA models in order to evaluate how Pyrodwarf and OHF microshoots respond to the mineral medium based on the number and length of new formed shoots obtained from the design of Box-behnken.
- Finding the optimal culture medium composition for maximizing the proliferation rate (PR), the average shoot length (SL), the quality index (QI), and minimizing shoot tip necrosis (STN) and vitrification (Vitri) by optimizing the obtained model using GA.

## Results

**Fig. 1:**
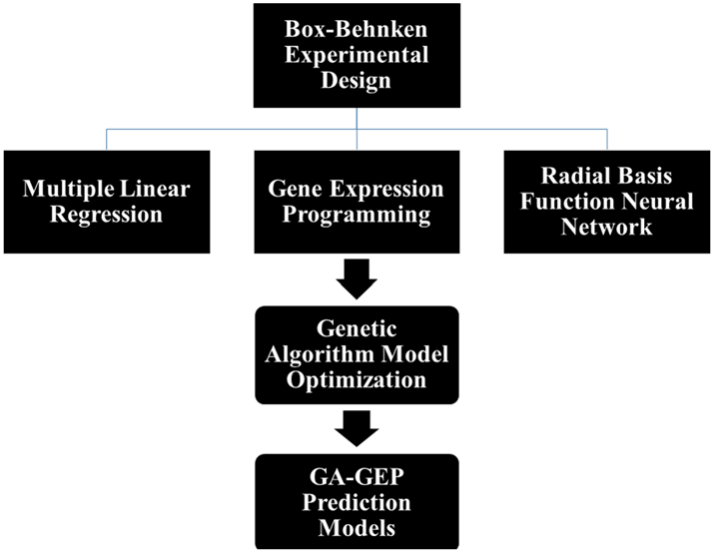
Framework of the performed experiments

This section evaluates the performances of the proposed models by analyzing the accuracy of each modelling approach for predicting the plant tissue culture media composition to studied fruit tree rootstocks micropropagation. Then GA-optimization of the most accurate modeling approach results is evaluated to achieve the most appropriate media compositions for each studied parameter. A framework of the performed experiments to achieve the best model is presented in Fig. 1.

### Comparison of MLR, RBFNN and GEP prediction models

The models were developed using MLR, RBFNN and GEP methods considering the media components variables that mainly affect the explant proliferation parameters. The MLR predictive model could not explain high variability of growth parameters due to the interaction of the variables considered, which may cover the media components effects. The mathematical equations obtained from GEP method that best approximate the growth parameters are given in Table 1. Moreover, the R^2^, RMSE, MARE and MBE statistics of each developed model are given in Table 2.

**Table 1:** Developed equations using gene expression programming for predicting explant growth parameters

Pear rootstock	Equation
Pyrodwarf	$PR = \left[ {\exp \left( {\left( {F - B} \right) \times c} \right) - D} \right] + \left[ {\left( {\ln \left( {\frac{{\sqrt {\frac{E - 2.1016}{2}} + E}}{2}} \right)} \right)^{ - 1} + D} \right] + \left[ {\left( {\sqrt[3]{3.1775 - C}} \right)^{2} + \sqrt[3]{{\frac{\exp \left( A \right)}{C + 0.11065}}}} \right]$
	$$SL = \left[ {\sqrt[3]{{{ \ln }\left( {D^{3} } \right)}} + \left( {F \times \left( {\frac{C + A}{2} \times E} \right)} \right)} \right] + \left[ {2.4011 \times \left( {\frac{{\left( {\frac{E + 1.7251}{2} \times 2.7057} \right) + \left( {1.7251 - B} \right)}}{2} - E} \right)^{2} } \right] + \left[ {\sqrt[3]{{2.3488 \times \left( {\left( {E^{ - 1} - 7.0907} \right) + \left( {E - D} \right)} \right)}}} \right]$$
	$$STN = \left[ {\frac{{D + \left( {\sqrt C \times \left( {F - E} \right)} \right)}}{2} + exp\left( {A - C} \right)} \right] + \left[ {\left( {B - \frac{{\frac{6.1753}{E} + E}}{2}} \right) + \left( {9.3446A \times { \ln }\left( B \right)} \right)} \right] + \left[ {\frac{{\left( {\left( {\ln \left( F \right) + \frac{11.3038}{A}} \right) - E + 1.0814} \right) + 15.0404A^{2} }}{2}} \right]$$
	$$VITRI = \left[ {\frac{{\left( {{ \exp }\left( B \right) \times B} \right) \times {\text{C}}^{ - 1} }}{2} - \left( {D - B} \right) - 1.5069} \right] + \left[ {\frac{{\exp \left( {2.988 - D} \right) + \frac{A - 9.0917}{2}}}{2} \times \left( {2B - \left( {B \times D} \right)} \right)} \right] + \left[ {\frac{{\frac{{\frac{E + B}{2} + B}}{2} + F + 8.8145}}{2} \times \left( {\frac{A + B}{2} + 2B} \right)} \right]$$
	$$QL = \left[ {\sqrt[9]{{\frac{D \times A}{\sqrt C \times B}}}} \right] + \left[ {\sqrt[3]{{\left( {\frac{{\frac{E}{30.7869} + B}}{2}} \right)^{ - 1} + \left( {0.9692 - A - B} \right)}}} \right] + \left[ {\sqrt[6]{{\left( {\exp \left( C \right) \times \left( {10.3785 + F} \right)} \right) + F^{ - 1} - A}}} \right]$$
OHF	
	$$PR = \left[ {\sqrt {\left( {0.0894D \times B} \right) + \left( {A + E} \right)^{ - 1} } } \right] + \left[ {\left( {\sqrt[3]{{\frac{ - 8.5762}{A} - 6.8234}}} \right) \times \left( {3.2661 - E} \right)^{ - 1} } \right] + \left[ {\frac{{\frac{C + 0.1838}{2} + 6.8196}}{2} + C^{ - 1} + \left( {\left( {B \times F} \right) - B} \right)} \right]$$
	$$SL = \left[ {\frac{{\ln \left( {\frac{B + 4.8333}{2}} \right) + \left( {\frac{D + 4.7651}{2} - \left( {A + B} \right)} \right)}}{2}} \right] + \left[ {C + \left( {{ \exp }\left( {\left( { - 2.8335A} \right) \times \left( {\frac{{\left( {D - 6.4679} \right) + \left( {9.3060C} \right)}}{2}} \right)} \right)} \right)} \right] + \left[ {{ \exp }\left( {\frac{{\left( {\left( {3.8362F} \right) - A} \right) + \left( {\frac{E + 3.1237}{2}} \right)}}{2} - \left( {3.4882 - E} \right)^{ - 1} } \right)} \right]$$
	$$STN = \left[ {B^{2} - \left( {\sqrt[3]{{\frac{{\frac{C}{E} + 8.1303}}{{{ \ln }\left( A \right)}}}}} \right)} \right] + \left[ {\exp \left( A \right) + C^{3} - C} \right] + \left[ {F + \frac{{D + \left( {\left( {\frac{ - 4.4380}{C}} \right)^{2} - \frac{6.1907}{B}} \right)}}{2}} \right]$$
	$$VITRI = \left[ {E \times \left( {A + \frac{{\left( {\frac{E - 0.8805}{2}} \right) \times B}}{F + A}} \right)} \right] + \left[ {\frac{{A - \left( {\sqrt[3]{A} - 6.8169} \right)}}{{D^{2} }}} \right] + \left[ {\left( {\frac{A + 2D - 6.8579}{2}} \right) \times \left( {\frac{{D^{2} + \frac{B}{A}}}{2}} \right)} \right]$$
	$$QL = \left[ {\sqrt {\left( {\sqrt[4]{{\left( {\frac{{E^{ - 1} + 8.3712}}{2}} \right) \times \left( {C - F} \right)}}} \right)^{3} } } \right] + \left[ {\left( {\left( {C - 5.1079} \right) \times \sqrt D } \right) \times \left( {\left( {C \times A} \right) - 5.0292} \right)} \right]^{ - 1} + \left[ {\frac{{B^{ - 1} + ln\left( {\left( {ln\frac{C}{0.3661}} \right) \times \left( {D + F} \right)} \right)}}{2}} \right]$$

A: NH_4_NO_3_, B: KNO_3_, C: mesos, D: minor, E: 6-benzylaminopurine (BA), F: indole-3-butyric acid (IBA)

**Table 2:** Comparison statistics on different constructed models using MLR, RBFNN and GEP techniques for PR, SL, STN, Vitri and QI of OHF and Pyrodwarf rootstocks during in vitro proliferation

Rootstock	Measured parameter	Model	RMSE	MARE	MBE	R^2^
OHF	PR	MLR	1.82	0.534	0.030	0.583
		RBFNN	0.69	0.138	− 0.014	0.957
		GEP	0.48	0.128	− 0.015	0.992
	SL	MLR	0.38	0.104	− 0.005	0.904
		RBFNN	0.14	0.034	0.012	0.990
		GEP	0.22	0.058	0.040	0.974
	STN	MLR	10.77	0.523	0.184	0.647
		RBFNN	6.95	0.211	0.118	0.882
		GEP	4.21	0.138	− 0.096	0.961
	Vitri	MLR	9.83	0.649	− 0.222	0.511
		RBFNN	5.27	0.169	− 0.351	0.883
		GEP	3.26	0.132	0.075	0.969
	QI	MLR	0.87	0.269	− 0.019	0.668
		RBFNN	0.52	0.155	− 0.028	0.891
		GEP	0.45	0.160	− 0.018	0.943
Pyrodwarf	PR	MLR	3.58	1.160	− 1.572	0.315
		RBFNN	1.49	0.379	0.087	0.908
		GEP	0.38	0.071	− 0.003	0.994
	SL	MLR	1.13	0.552	0.011	0.375
		RBFNN	0.43	0.194	− 0.007	0.924
		GEP	0.30	0.130	0.009	0.978
	STN	MLR	6.48	0.217	− 0.438	0.855
		RBFNN	5.42	0.158	− 0.456	0.906
		GEP	4.22	0.126	0.045	0.954
	Vitri	MLR	5.86	0.193	0.915	0.909
		RBFNN	5.56	0.158	− 0.499	0.901
		GEP	4.59	0.142	− 0.496	0.936
	QI	MLR	0.47	0.171	− 0.063	0.902
		RBFNN	0.43	0.163	0.032	0.923
		GEP	0.41	0.160	− 0.018	0.944

Results of the calculated statistics for output variables (PR, SL, Vitri, STN and QI) corresponding to the GEP and RBFNN showed a substantially higher accuracy of prediction than for MLR models. As, the amounts of calculated R^2^ for GEP and RBFNN vs. MLR models were: PR = 0.992 and 0.957 vs. 0.583, SL = 0.974 and 0.990 vs. 0.904, STN = 0.961and 0.882 vs. 0.674, Vitri = 0.969 and 0.883 vs. 0.511 and QI = 0.943 and 0.891 vs. 0.668 in OHF and PR = 0.994 and 0.908 vs. 0.315, SL = 0.978 and 0.924 vs. 0.357, STN = 0.954 and 0.906 vs. 0.855, Vitri = 0.936 and 0.901 vs. 0.909 and QI = 0.944 and 0.923 vs. 0.902 in Pyrodwarf, respectively (Table 2).

Comparing the accuracy results of GEP and RBFNN (Table 2) showed that GEP is more accurate in case of all studied parameters in both rootstocks except SL in OHF (R^2^ = 0.974 and 0.990, RMSE = 0.14 and 0.22, MARE = 0.034 and 0.058 and MBE = 0.012 and 0.040 for RBFNN and GEP, respectively). Therefore, to find the optimized medium for achieving the highest quality and quantity in proliferation, we optimized constructed GEP models using GA technique.

### GEP models optimization

Finally, the GEP models were analyzed to find the optimum levels of NH_4_NO_3_, KNO_3_, mesos nutrients, micro-nutrients and 6-benzylaminopurine (BA) and indole-3-butyric acid (IBA) hormones to be used in the culture medium for achieving the maximum PR, SL and QI, as well as the minimum STN and Vitri occurrence in *Pyrus* rootstocks. The predicted values and the optimization of studied growth parameters by the GEP models are presented in Table 3.

**Table 3:** Optimization analysis on GEP models to achieve maximum PR, SL and QI and minimum STN and Vitri in OHF and Pyrodwarf pear rootstocks

*Pyrus* rootstock	NH_4_NO_3_	KNO_3_	Mesos	Minor	BA	IBA
Pyrodwarf						
PR						
13.00	1.56	1.19	1.75	3.28	2.08	0.13
SL						
5.00	1.83	0.51	1.74	2.60	0.59	0.16
STN						
0.00	0.93	0.50	2.30	0.64	0.51	0.07
Vitri						
4.00	0.50	0.50	2.50	2.76	0.50	0.05
QI						
4.99	0.89	0.50	2.50	4.00	0.50	0.05
OHF						
PR						
9.42	2.00	1.19	0.62	2.13	1.59	0.17
SL						
4.99	0.90	1.35	2.04	3.45	1.81	0.10
STN						
0.02	1.08	0.51	1.58	2.36	1.93	0.15
Vitri						
0.02	0.50	1.27	1.49	1.52	1.32	0.14
QI						
4.84	0.99	0.53	1.86	2.12	2.59	0.18

Comparing observed and predicted outputs showed the behavior of the constructed models from investigating inputs. The GA optimization analysis on the GEP model showed that media containing 1.56 NH_4_NO_3_, 1.19 KNO_3_, 1.75 mesos nutrients, 3.28 micro-nutrients, 2.08 BA and 0.13 IBA could lead to optimal PR in Pyrodwarf (13.0) and media containing 2.00 NH_4_NO_3_, 1.19 KNO_3_, 0.62 mesos nutrients, 2.13 micro-nutrients, 1.59 BA and 0.17 IBA resulted in optimal PR in OHF (9.4). The optimal SL in pyrodwarf (5.0 cm) could be obtained with media comprising 1.83 NH_4_NO_3_, 0.51 KNO_3_, 1.74 mesos nutrients, 2.60 micro-nutrients, 0.60 BA and 0.16 IBA and in OHF (5.0) with media containing 0.90 NH_4_NO_3_, 1.35 KNO_3_, 2.04 mesos nutrients, 3.45 micro-nutrients, 1.81 BA and 0.10 IBA. Results of our study exhibited that media containing 0.93 NH_4_NO_3_, 0.50 KNO_3_, 2.30 mesos nutrients, 0.64 micro-nutrients, 0.51 BA and 0.07 IBA and 1.08 NH_4_NO_3_, 0.51 KNO_3_, 1.58 mesos nutrients, 2.36 micro-nutrients, 1.93 BA and 0.15 IBA could lead to minimum STN in pyrodwarf (0.00) and OHF (0.02), respectively. In Pyrodwarf rootstock (4.0), media with 0.50 NH_4_NO_3_, 0.50 KNO_3_, 2.50 mesos nutrients, 2.76 micro-nutrients, 0.50 BA and 0.05 IBA could result in the optimal point for Vitri, whereas in OHF rootstock (0.02) the optimal point for Vitri could be achieved with media supplemented with 0.50 NH_4_NO_3_, 1.27 KNO_3_, 1.49 mesos nutrients, 1.52 micro-nutrients, 1.31 BA and 0.14 IBA. Lastly, the optimization results of RBFNN models revealed that media containing 0.89 NH_4_NO_3_, 0.50 KNO_3_, 2.50 mesos nutrients, 4.0 micro-nutrients, 0.50 BA and 0.05 IBA in Pyrodwarf (4.99) and 0.99 NH_4_NO_3_, 0.53 KNO_3_, 1.88 mesos nutrients, 2.12 micro-nutrients, 2.59 BA and 0.18 IBA in OHF could produce optimal explant quality.

The media components suggested by the optimized GEP model revealed that although decrease in NH_4_NO_3_, KNO_3_ and micro-nutrients and BA and IBA hormones and increase in mesos nutrients may reduce PR but it also may reduce STN and Vitri occurrences which cause the higher QI of in vitro multiplied Pyrodwarf rootstock. But it was a little different for OHF rootstock as decrease in NH_4_NO_3_ and KNO_3_ and increase in mesos nutrients and BA hormone caused lower STN and higher QI, but decrease in KNO_3_ was not effective on Vitri reduction. The positive effect of reduction in NO_3_^−^ and NH_4_^+^ concentrations on PR has also been previously reported [15] on G × N15 rootstock. In addition, the effective role of higher concentration of some mesos components like CaCl_2_ has also been stated b by [47].

## Discussion

AI has recently been successfully and progressively applied in plant bio-researches [48] as well as for predicting the optimal plant tissue culture conditions [49] and media components [10, 15, 50, 51]. The in vitro plant tissues development is under control of the culture media nutrients. As the optimization of the media mineral composition is a laborious and time-consuming job, so predicting the favorable growth media composition is very helpful for achieving maximum efficiency. Developing neural models to investigate the effect of sucrose and light on the in vitro proliferation of kiwifruit (*Actinidia deliciosa*) [31] and the effect of macronutrients content in the culture media on the G × N15 explant growth [15] are some examples. In our previous work on pear rootstocks (OHF and Pyrodwarf), we modeled the effect of the eight macronutrients concentrations on explant growth parameters by evaluating the optimal structure of the multilayer perceptron neural network (MLPNN) [10]. The mentioned study showed that ANN-based analyses are promising in predicting the required macronutrients concentrations for maximizing the explant PR and SL and minimizing the explant STN, chlorosis, and Vitri which was not possible using linear regression analysis [10].

In the present study, we used RBFNN as a more robust predicting tool than MLPNN due to working as an interpolator in multidimensional space whereas MLPNN works in stochastic manner. In the other words, RBFNN is more ‘function approximator’ than ‘pattern recognizer’ whilst MLPNN is the contrary [52]. GEP also was used as an expansion of GA and GP to predict the relationship between input and output. The MLR was also used to compare the power of new above mentioned methods in predicting the optimal points of plant tissue culture media components for achieving the most appropriate results in proliferation of *Pyrus* rootstocks.

The main advantage of ANN-based models is that they do not require a preceding specification of appropriate fitting function so; they have a general approximation capability to estimate practically all types of non-linear functions. This flexibility characteristic may benefit the modeler to construct a model with the highest probable prediction accuracy.

One of the most important advantages of using GP-based methods like GEP, over other approaches is their ability for production of prediction equations without any assumption for prior form of the relationship. GP as well as its variants have been employed by many researchers to discover any complex relationships which fit various experimental data [53–55]. A population of individuals is used in this method and then, better individuals are selected by employing genetic variations and fitness function. Genetic operators introduce the genetic variations. GEP as a learning machine is supposed to learn the relationship between variables in groups of data. The difference between GEP and its precursors GP and GA is in the way of individual programming as in GEP, individuals are programmed as fixed length linear strings (chromosomes) which are shown eventually by expression trees which are a simple diagram presentation. While, individuals are expressed in GP and GA as fixed length linear strings (chromosomes) and nonlinear entities of different shapes (parse trees) and sizes, respectively. Genetic operators work at the chromosome level in GEP making creation of genetic diversity very simple, which is considered as one of its strengths over GP and GA. The unique, multi-genic nature of GEP is its other significant point allowing more complex programs with several sub-programs to be evolved. The benefits of both GP and GA are combined in GEP, while some of their limitations are met [39].

Our previous results on the same *Pyrus* rootstocks showed that the performance of explant in response to macronutrients concentrations varies according to the pear rootstock genotype, as well. So that, NO_3_^−^ was found to be critical for OHF explant while NH_4_^+^ was found to be critical nutrient for Pyrodwarf explant growth and therefore, we suggested that the use of ANN-based model analyses would lead us to detect the optimized macronutrient concentrations essential to maximize the PR and SL and minimize the occurrence of STN and Vitri [10]. Due to the complicated interactions between medium components, determining minerals and hormones optimum levels for a special genotype is very difficult [56]. Moreover, different media are needed for optimal growth of *Pyrus* genotypes because of the incidence of the physiological disorders like hyperhydricity and necrosis at the explant multiplication stage. In order to design an optimized culture medium, use of a trustworthy mathematical modeling and optimization technique is necessary to achieve an optimal and efficient growth [10, 15, 50, 51]. Diverse statistical techniques have been used previously to design new and effective plant tissue culture media [10, 15, 32, 50, 51, 57]. RSM and MLR have been constantly applied to optimize new in vitro media for pear genotypes [10, 19]. The studies reported that ANN-GA models had a significantly higher accuracy of prediction than RSM and MLR [10, 58]. It has been reported that RSM and MLR alone could not be reliable methods for estimating non-polynomial or non-linear relationships among variables [10, 59].

Briefly, we found different RBFNN-based models with optimization algorithm to integrate the obtained data set of the in vitro responses of *Pyrus* rootstocks to nutrients and hormones concentrations. These optimized models could reveal the importance of each studied nutrient or hormone in increasing or decreasing each studied feature. Previous studies using ANN-GA models on G × N15 *Prunus* rootstock from a gathered data proposed that the role of NH_4_^+^, NO_3_^−^, PO_4_^2−^, Ca^2+^, and K^+^ were more important than SO_4_^2−^, Mg^2+^, and Cl^−^ in in vitro proliferation [15]. Here, we found from the GEP optimized models that increasing proliferation may lead to less plantlet quality. So, we did GEP analysis to find the best medium resulting to high quality proliferated plantlets and lowest losses.

NH_4_NO_3_ and KNO_3_ are the major sources of nitrogen and potassium for *Pyrus* rootstocks micropropagation. The importance of the ratio between NO_3_^−^ and NH_4_^+^ concentrations has been widely discussed in literatures [60–63]. The present results are in coincidence with many previous reports on different plant species [60, 61, 64, 65]. The medium macro-nutrients contents are major determinants of the explant growth responses. The pear rootstock responses to in vitro nutrients has been reported that varies with the macro-nutrients levels in the culture medium [10]. Comparison of our comprehensive results to the previous results [10, 15, 50, 51] showed for the first time that the concentrations of macro- and micro-nutrients depend highly on the concentrations of used hormones as their interaction could determine the quality of plantlets. Arab et al. [15] predicted and maximized the number and length of in vitro regenerated shoots by decreasing NH_4_^+^ concentration and optimizing NO_3_^−^ concentration, simultaneously. But they predicted that enhancing the NH_4_^+^ concentration will increase SL while producing non-healthy shoots while reducing its amount will increase the quality of plantlets. It has been suggested that an optimized culture medium would produce a higher number and length of shoots. Andreu and Marín [66] reported that reducing nitrogen content had a proper effect on proliferation rate. Our results using optimized RBFNN and GEP modeling (Table 3) also showed that a lower nitrogen concentration content in the medium will produce higher quality plantlets which is consistent with the previous results [60, 67, 68]. The purpose of our recent studies [10, 15, 50, 51] was to present a more and more precise approach for prediction of an optimized culture medium. Here, techniques of RBFNN and GEP combined with GA were applied to pear rootstocks proliferation experiment data sets for finding the best proliferation results. Comparing the present results with the previous ones [10, 15, 50, 51] shows that using both methods together leads us to more accurate consequences. As, comparing these two techniques results showed the effect of each medium component increasing or decreasing the measured parameter (Tables 2, 3).

Interactions between plant hormones make a critical complexity in regulating plant growth processes. Cytokinin has been shown that regulates cell proliferation [69] and auxin increases the sensitivity of the less mitotically active cells of apical meristem to cytokinin [70]. The ratio of cytokinin to auxin is a crucial signal which determines phenotype [71]. The effects of hormones depend on plant species. The results of [50] on *Prunus* rootstock showed that using cytokinin and auxin together in the medium will make higher shooting than using each alone. They indicated that the concentration and interaction of hormones are two important factors on in vitro shooting. In accordance with these results and the findings of [11, 72], we used different concentrations of BA and IBA in our protocol formulation. As auxin and cytokinin have roles in DNA replication and cell cycle regulation, respectively [73]. The adverse results of [50] was reported to be due to the interaction of many factors like genotype and the composition of culture medium [74] with hormones. So, in the present study, we assessed the interaction of hormones and medium nutrients on proliferation. Since the high concentration of hormonal combinations may result in low SL and regenerated shoots quality and low concentrations of auxin may prompt cell division but its higher concentrations may inhibit axillary bud growth [75], we considered BA and IBA concentrations in a reasonable range to achieve the most effective protocol. Our experiments analyses using GA optimized GEP modeling procedure showed that this technique can be used as an effective method for studying the interaction of many factors on growth parameters in proliferation stage. So, GEP is introduced for the first time as a great tool in optimizing higher quality and efficiency plant tissue culture protocols in less time.

In general, the use of ANN-based models such as RBFNN leads to the high accuracy of estimation whereas GEP models are easier in the use to give it an explicit mathematical equation in prediction of explant growth parameters as well as being more accurate than RBFNN method. No studies have been done on the superiority of RBFNN or GEP approaches for estimating in vitro explant proliferation parameters with optimal use of media components. Therefore, according to the current results, the approaches presented here would allow more accurate estimations without the need for the availability of all data.

## Conclusions

The main objective of this paper was to compare the performance of MLR, RBFNN and GEP models for predicting the concentrations of in vitro medium components to achieve the optimal growth parameters. Different combinations of the nutrients and hormones were used as inputs for the MLR, RBFNN and GEP techniques. The proliferation parameters were estimated from the GEP equations. PR, SL, Vitri, STN and QI were chosen as the main indices of proliferation state. Our results suggested that using RBFNN and GEP techniques leads to more accurate results. The optimized GEP models gave us the most appropriate formulation for each studied parameter so, these results can cause finding the most efficient protocol. The GEP constructed models were the most accurate models as well as being easier to apply, as they estimate growth parameters using explicit statistical equations. Using further optimization techniques are suggested for future studies on predicting and optimizing plant tissue culture media by GEP modeling procedure to achieve the most appropriate results.

## Methods

MLR, RBFNN and GEP modeling procedures were used to construct models by using different combinations of minerals and hormones concentrations as inputs and various measured proliferation parameters as outputs. The GEP models were applied to find the optimized models using GA. Two case studies were performed using two pear rootstocks which have described details of the used techniques to realize the optimized inputs compositions as follows.

### Case studies

Pear rootstocks Pyrodwarf (“Old Home” × “Gute Luise”) and OHF (“OldHome” × “Farmingdel”) from in vitro cultures were grown in modified macro- and micro-nutrient MS media supplemented with 30 g/l sucrose, 8 g/l agar (DuchefaH) and different concentrations of cytokinin and auxin hormones. Media pH was adjusted to 5.7, distributed into 250 ml jam jars with polyethylene caps and autoclaved at 1 kg cm^−2^ s^−1^ (121 °C) for 15 min. All cultures were incubated at 25 ± 2 °C with a 16-h light period (80 µmol m^2^ s^−1^) of white fluorescent light for 4 weeks. Afterwards, variables including PR, SL, Vit, STN and QI were recorded. In each set of experiments, each treatment consisted of 10 replicates (jam jars) and each replication included four explants for both Pyrodwarf and OHF rootstocks.

### Box–Behnken experimental design as an invaluable tool for optimization of explant growth parameters

In recent years, multivariate experimental designs such as Central Composition and Box–Behnken designs (BBD) have been frequently used for evaluating the effects of variables in many processes. The most important advantage of these designs is decrease in the number of experiments essential to conduct the studies, which results in saving time and cost as well as a decrease in materials and reagents consumption. Additionally, the statistical analysis carried out on the results is quickly realized and the percentage amount of experimental error is diminished [76–79]. In the present investigation, Box–Behnken statistical screening design with 6 variables, 3 levels and 48 runs was applied to assess accurately the main and quadratic effects of independent variables on dependent variables. BBD is a useful and formidably effective tool for optimizing process in a way that variables and their interactions could be recognized with a minimum in the number of experimental trials [79, 80]. It is a very complicated design with a practical combination of two-level factorial designs with incomplete block designs [79, 81, 82]. Furthermore, previous researches have demonstrated that BBD is more powerful and efficient than the three-level factorial and Central Composition designs [83, 84].

In the present experiment, KNO_3_, NH_4_NO_3_, Mesos, minors, BAP and IBA were determined as independent (input) variables and PR, SL, Vit, STN and QI were selected as dependent (target) variables. Different concentrations of independent variables were determined based on their content in MS medium (1 × MS concentration) (Table 4).

**Table 4:** The factor components and range of experimental runs according to MS basal medium

Variables	Components	Range
Factor 1	KNO_3_	0.5–2×
Factor 2	NH_4_NO_3_	0.5–2×
Factor 3 (mesos)	CaCl_2_ KH_2_PO_4_ MgSO_4_	0.5–2.5×
Factor 4 (minors)	CoCl_2_·6H_2_O CuSO_4_·5H_2_O H_3_BO_3_ Kl MnSO_4_·H_2_O Na_2_MoO_4_·2H_2_O ZnSO_4_·7H_2_O FeNaEDTA	0.5–4×
Factor 5 (hormone)	BAP	0.5–2.5 mg l^−1^
Factor 6 (hormone)	IBA	0.05–0.2 mg l^−1^

After establishing the range of independent variables at three levels in a coded form: − 1 (low), 0 (mid) and + 1 (high) (Table 5), the experimental trials based on a BBD were set up (Table 6). The actual values and observed results for the three levels of the variables studied are presented in Tables 7 and 8.

**Table 5:** level of factors according to Box–Behnken Design

Factors	Coded variable level		
	Low	Mid	High
	− 1	0	1
KNO_3_	0.5	1.25	2
NH_4_NO_3_	0.5	1.25	2
Mesos	0.5	1.5	2.5
Minors	0.5	2.25	4
BAP	0.5	1.75	3
IBA	0.05	0.13	0.2

**Table 6:** Level of factors according to Box–Behnken design

Culture medium	Level of factors used in each experiment					
	KNO_3_	NH_4_NO_3_	Mesos	Minors	BAP	IBA
1	1.25	0.50	1.50	2.25	0.50	0.20
2	2.00	2.00	1.50	0.50	1.75	0.13
3	1.25	0.50	2.50	2.25	0.50	0.13
4	2.00	1.25	0.50	2.25	1.75	0.05
5	2.00	1.25	2.50	2.25	1.75	0.05
6	2.00	1.25	1.50	0.50	3.00	0.13
7	1.25	0.50	1.50	2.25	3.00	0.05
8	1.25	1.25	0.50	4.00	1.75	0.05
9	2.00	1.25	2.50	2.25	1.75	0.20
10	1.25	2.00	1.50	2.25	3.00	0.20
11	1.25	1.25	2.50	4.00	1.75	0.20
12	0.50	0.50	1.50	4.00	1.75	0.13
13	2.00	0.50	1.50	4.00	1.75	0.13
14	1.25	2.00	0.50	2.25	3.00	0.13
15	0.50	1.25	0.50	2.25	1.75	0.05
16	2.00	0.50	1.50	0.50	1.75	0.13
17	1.25	0.50	1.50	2.25	3.00	0.20
18	1.25	1.25	2.50	0.50	1.75	0.20
19	1.25	0.50	1.50	2.25	0.50	0.05
20	1.25	2.00	2.50	2.25	3.00	0.13
21	1.25	0.50	0.50	2.25	0.50	0.13
22	2.00	1.25	0.50	2.25	1.75	0.20
23	1.25	2.00	0.50	2.25	0.50	0.13
24	1.25	0.50	0.50	2.25	3.00	0.13
25	1.25	2.00	1.50	2.25	3.00	0.05
26	0.50	0.50	1.50	0.50	1.75	0.13
27	0.50	1.25	1.50	4.00	0.50	0.13
28	0.50	1.25	2.50	2.25	1.75	0.20
29	2.00	1.25	1.50	4.00	3.00	0.13
30	1.25	1.25	0.50	0.50	1.75	0.20
31	0.50	2.00	1.50	0.50	1.75	0.13
32	1.25	1.25	2.50	0.50	1.75	0.05
33	0.50	1.25	1.50	0.50	3.00	0.13
34	1.25	1.25	2.50	4.00	1.75	0.05
35	2.00	1.25	1.50	4.00	0.50	0.13
36	1.25	1.25	0.50	0.50	1.75	0.05
37	2.00	1.25	1.50	0.50	0.50	0.13
38	1.25	2.00	1.50	2.25	0.50	0.20
39	1.25	2.00	1.50	2.25	0.50	0.05
40	0.50	1.25	2.50	2.25	1.75	0.05
41	2.00	2.00	1.50	4.00	1.75	0.13
42	1.25	0.50	2.50	2.25	3.00	0.13
43	1.25	1.25	0.50	4.00	1.75	0.20
44	0.50	1.25	1.50	4.00	3.00	0.13
45	1.25	2.00	2.50	2.25	0.50	0.13
46	0.50	1.25	0.50	2.25	1.75	0.20
47	0.50	2.00	1.50	4.00	1.75	0.13
48	0.50	1.25	1.50	0.50	0.50	0.13

**Table 7:** Box–Behnken design of OHF micropropagation experiments and average values of the parameters used to characterize it

Culture medium	Factor 1	Factor 2	Factor 3	Factor 4	Factor 5 (mgl^−1^)	Factor 6 (mgl^−1^)	PR	SL (cm)	STN	Vitri	QL
	KNO_3_	NH_4_NO_3_	Mesos	Minors	BAP	IBA					
1	1.25	0.50	1.50	2.25	0.50	0.20	2.00	4.51	0	0	5.00
2	2.00	2.00	1.50	0.50	1.75	0.13	6.75	2.52	14.80	33.33	2.00
3	1.25	0.50	2.50	2.25	0.50	0.13	1.00	4.96	0	0	5.00
4	2.00	1.25	0.50	2.25	1.75	0.05	8.50	2.22	44.10	8.82	1.50
5	2.00	1.25	2.50	2.25	1.75	0.05	5.50	3.74	18.18	4.55	3.75
6	2.00	1.25	1.50	0.50	3.00	0.13	4.50	2.25	11.11	38.85	1.75
7	1.25	0.50	1.50	2.25	3.00	0.05	4.75	3.34	0	5.26	4.75
8	1.25	1.25	0.50	4.00	1.75	0.05	7.75	3.00	38.70	16.13	1.50
9	2.00	1.25	2.50	2.25	1.75	0.20	5.75	4.07	21.73	4.35	3.50
10	1.25	2.00	1.50	2.25	3.00	0.20	4.25	2.91	11.76	5.88	3.75
11	1.25	1.25	2.50	4.00	1.75	0.20	5.50	5.57	13.62	13.64	3.50
12	0.50	0.50	1.50	4.00	1.75	0.13	6.25	4.80	8.00	8.00	4.25
13	2.00	0.50	1.50	4.00	1.75	0.13	7.25	3.71	10.32	13.79	3.75
14	1.25	2.00	0.50	2.25	3.00	0.13	5.25	1.69	47.61	9.52	1.25
15	0.50	1.25	0.50	2.25	1.75	0.05	7.25	2.82	41.37	0	1.75
16	2.00	0.50	1.50	0.50	1.75	0.13	7.25	3.20	6.88	31.03	2.25
17	1.25	0.50	1.50	2.25	3.00	0.20	4.75	3.49	0	5.26	4.75
18	1.25	1.25	2.50	0.50	1.75	0.20	5.50	4.04	13.62	27.27	2.50
19	1.25	0.50	1.50	2.25	0.50	0.05	2.00	3.89	0	0	5.00
20	1.25	2.00	2.50	2.25	3.00	0.13	3.00	3.00	24.99	8.33	3.25
21	1.25	0.50	0.50	2.25	0.50	0.13	2.75	2.68	36.36	0	2.00
22	2.00	1.25	0.50	2.25	1.75	0.20	8.75	2.37	45.71	8.57	1.25
23	1.25	2.00	0.50	2.25	0.50	0.13	2.25	2.15	44.44	0	1.75
24	1.25	0.50	0.50	2.25	3.00	0.13	6.00	2.00	29.16	4.17	3.00
25	1.25	2.00	1.50	2.25	3.00	0.05	4.25	2.60	11.76	5.88	3.75
26	0.50	0.50	1.50	0.50	1.75	0.13	6.25	3.28	4.00	24.00	3.25
27	0.50	1.25	1.50	4.00	0.50	0.13	1.25	4.17	20.00	20.00	2.75
28	0.50	1.25	2.50	2.25	1.75	0.20	5.25	4.50	19.04	0	4.00
29	2.00	1.25	1.50	4.00	3.00	0.13	4.50	2.65	11.10	22.20	2.75
30	1.25	1.25	0.50	0.50	1.75	0.20	7.75	2.50	41.86	32.25	1.00
31	0.50	2.00	1.50	0.50	1.75	0.13	6.00	2.71	8.33	25.00	3.25
32	1.25	1.25	2.50	0.50	1.75	0.05	5.50	3.90	13.62	31.82	2.00
33	0.50	1.25	1.50	0.50	3.00	0.13	4.00	2.02	6.25	31.25	2.50
34	1.25	1.25	2.50	4.00	1.75	0.05	5.25	5.00	14.28	13.83	3.50
35	2.00	1.25	1.50	4.00	0.50	0.13	1.75	3.07	14.28	14.29	3.50
36	1.25	1.25	0.50	0.50	1.75	0.05	7.75	2.20	38.64	29.03	1.75
37	2.00	1.25	1.50	0.50	0.50	0.13	1.75	2.31	14.28	42.86	1.25
38	1.25	2.00	1.50	2.25	0.50	0.20	1.50	3.88	16.66	0	3.75
39	1.25	2.00	1.50	2.25	0.50	0.05	1.50	3.36	16.66	0	3.75
40	0.50	1.25	2.50	2.25	1.75	0.05	5.25	4.27	19.04	0	3.50
41	2.00	2.00	1.50	4.00	1.75	0.13	6.75	2.75	18.50	18.52	3.25
42	1.25	0.50	2.50	2.25	3.00	0.13	3.75	3.50	13.32	0	3.75
43	1.25	1.25	0.50	4.00	1.75	0.20	8.00	3.39	43.75	12.50	1.25
44	0.50	1.25	1.50	4.00	3.00	0.13	4.00	2.90	12.50	18.75	3.50
45	1.25	2.00	2.50	2.25	0.50	0.13	1.00	4.01	25.00	0	3.00
46	0.50	1.25	0.50	2.25	1.75	0.20	7.50	3.33	39.99	0	2.50
47	0.50	2.00	1.50	4.00	1.75	0.13	6.00	4.29	12.48	8.33	3.75
48	0.50	1.25	1.50	0.50	0.50	0.13	1.25	3.04	0	20.00	3.00
MS	1.00	1.00	1.00	1.00	2.50	0.20	3.25	3.08	7.69	0	4.25
WPM	1.00	1.00	1.00	1.00	2.50	0.20	2.25	4.25	11.11	22.22	3.00
QL	1.00	1.00	1.00	1.00	2.50	0.20	3.00	2.81	0	0	5.00

**Table 8:** Box–Behnken design of Pyrodwarf micropropagation experiments and average values of the parameters used to characterize it

Culture medium	Factor 1	Factor 2	Factor 3	Factor 4	Factor 5 (mgl^−1^)	Factor 6 (mgl^−1^)	PR	SL (cm)	STN	Vitri	QL
	KNO_3_	NH_4_NO_3_	Mesos	Minors	BAP	IBA					
1	1.25	0.50	1.50	2.25	0.50	0.20	2.43	5.32	0	5.33	4.68
2	2.00	2.00	1.50	0.50	1.75	0.13	7.45	1.23	40.17	49.64	1.00
3	1.25	0.50	2.50	2.25	0.50	0.13	1.78	4.93	0	6.43	4.45
4	2.00	1.25	0.50	2.25	1.75	0.05	8.98	2.46	37.32	21.97	1.75
5	2.00	1.25	2.50	2.25	1.75	0.05	8.18	2.51	36.09	21.40	1.78
6	2.00	1.25	1.50	0.50	3.00	0.13	5.13	0.96	31.68	30.25	1.50
7	1.25	0.50	1.50	2.25	3.00	0.05	6.33	3.77	0	5.10	4.53
8	1.25	1.25	0.50	4.00	1.75	0.05	10.18	0.81	17.44	23.35	2.80
9	2.00	1.25	2.50	2.25	1.75	0.20	8.50	2.75	37.14	21.48	1.85
10	1.25	2.00	1.50	2.25	3.00	0.20	5.55	3.47	22.51	37.84	1.65
11	1.25	1.25	2.50	4.00	1.75	0.20	9.33	1.58	16.09	23.04	2.80
12	0.50	0.50	1.50	4.00	1.75	0.13	10.73	1.51	10.01	7.43	3.88
13	2.00	0.50	1.50	4.00	1.75	0.13	9.03	1.77	20.20	9.19	3.48
14	1.25	2.00	0.50	2.25	3.00	0.13	6.00	3.15	24.95	39.17	1.45
15	0.50	1.25	0.50	2.25	1.75	0.05	11.73	2.25	10.28	17.69	3.58
16	2.00	0.50	1.50	0.50	1.75	0.13	9.03	2.08	19.11	12.96	3.30
17	1.25	0.50	1.50	2.25	3.00	0.20	6.35	4.25	0	5.88	4.43
18	1.25	1.25	2.50	0.50	1.75	0.20	9.18	1.85	14.36	28.47	2.58
19	1.25	0.50	1.50	2.25	0.50	0.05	2.23	4.81	0	4.86	4.60
20	1.25	2.00	2.50	2.25	3.00	0.13	5.10	3.15	24.02	38.25	2.68
21	1.25	0.50	0.50	2.25	0.50	0.13	3.08	4.63	3.99	6.76	4.23
22	2.00	1.25	0.50	2.25	1.75	0.20	9.15	2.85	38.00	22.15	1.68
23	1.25	2.00	0.50	2.25	0.50	0.13	2.10	3.07	25.16	38.29	1.53
24	1.25	0.50	0.50	2.25	3.00	0.13	7.03	3.54	5.04	7.05	4.13
25	1.25	2.00	1.50	2.25	3.00	0.05	5.38	3.10	20.51	38.11	1.70
26	0.50	0.50	1.50	0.50	1.75	0.13	10.55	1.83	9.27	10.18	3.83
27	0.50	1.25	1.50	4.00	0.50	0.13	1.80	1.34	8.45	19.23	3.35
28	0.50	1.25	2.50	2.25	1.75	0.20	9.80	2.44	10.46	17.34	3.45
29	2.00	1.25	1.50	4.00	3.00	0.13	4.80	0.91	34.91	23.90	1.75
30	1.25	1.25	0.50	0.50	1.75	0.20	10.08	1.36	17.41	29.10	2.28
31	0.50	2.00	1.50	0.50	1.75	0.13	9.40	0.99	13.11	45.71	1.45
32	1.25	1.25	2.50	0.50	1.75	0.05	9.13	1.53	14.78	28.22	2.53
33	0.50	1.25	1.50	0.50	3.00	0.13	5.75	1.25	7.40	25.21	3.13
34	1.25	1.25	2.50	4.00	1.75	0.05	9.23	1.32	14.09	22.74	2.70
35	2.00	1.25	1.50	4.00	0.50	0.13	1.15	1.32	35.00	23.00	1.65
36	1.25	1.25	0.50	0.50	1.75	0.05	9.95	1.12	16.58	28.65	2.38
37	2.00	1.25	1.50	0.50	0.50	0.13	1.00	1.55	32.50	30.00	1.78
38	1.25	2.00	1.50	2.25	0.50	0.20	1.20	3.14	23.00	36.50	1.70
39	1.25	2.00	1.50	2.25	0.50	0.05	1.03	2.95	19.50	36.50	1.80
40	0.50	1.25	2.50	2.25	1.75	0.05	9.75	2.04	9.23	17.16	3.50
41	2.00	2.00	1.50	4.00	1.75	0.13	7.78	1.04	41.00	43.23	1.00
42	1.25	0.50	2.50	2.25	3.00	0.13	5.60	3.84	0	6.72	4.45
43	1.25	1.25	0.50	4.00	1.75	0.20	10.38	1.06	19.04	23.13	2.65
44	0.50	1.25	1.50	4.00	3.00	0.13	5.83	0.84	8.60	19.72	3.28
45	1.25	2.00	2.50	2.25	0.50	0.13	1.00	3.23	25.00	37.50	1.63
46	0.50	1.25	0.50	2.25	1.75	0.20	12.08	2.25	12.00	17.80	3.48
47	0.50	2.00	1.50	4.00	1.75	0.13	9.50	0.76	15.56	40.25	1.63
48	0.50	1.25	1.50	0.50	0.50	0.13	1.63	1.64	7.20	26.07	2.95
MS	1.00	1.00	1.00	1.00	2.50	0.20	3.53	3.99	14.23	10.23	3.60
WPM	1.00	1.00	1.00	1.00	2.50	0.20	2.28	2.57	6.47	3.25	4.18
QL	1.00	1.00	1.00	1.00	2.50	0.20	2.95	2.62	2.73	0	4.78

At least 10 replicates were used for each experimental run. 357 experimental sets (70% of all available patterns) were randomly selected among 510 sets in order to train the modeling procedures and the other 153 sets (30% of all patterns) were used to test the models generalization capacity.

### Modeling systems

#### Multiple linear regression

MLR analysis is considered as a multivariate statistical technique to analyze the relationship between a dependent single variable and a group of independent variables. Prediction and explanation are two main objectives of MLR. The prediction of MLR involves the extent to which the dependent variables can be predicted by the independent variables. The explanation of MLR evaluates the regression coefficients, their sign, statistical interface and magnitude, for each independent variable [85].

Linear regression is characterized as the oldest statistical technique in regression and it is thought to be a benchmark method to be employed by new techniques. Like other regression techniques, MLR models the relationships between two or more independent variables and a response variable; this is accomplished a linear equation fitted to the observed data. In this approach, every value assigned to the independent variable k is associated with a value assigned to the dependent variable M. The regression line of population for n input variables × 1, × 2, …, kn is as follows:

1 $${\text{M }} = \, \alpha 0 \, + \, \alpha 1 {\text{k1 }} + \cdots + \, \alpha {\text{n}}$$

M is the dependent variable, α0 denotes a constant named intercept, k = (× 1, …, kn) represents an input variables vector and α denotes the regression coefficients vector, each of which belongs for each explanatory variable. The observed values of Y have different means and are assumed with the equal standard deviation ε. The software package of SPSS 19 was utilized in the MLR model.

### Radial basis function neural network

The RBFNN is able to simulate the brain of human to accomplish several different tasks easily. It can also crystallize the complex relation between the inputs and outputs using a fitting process which leads it to an approximation of any nonlinear function [35, 36, 86, 87].

The RBFNN is comprised of three different layers: an input layer, a hidden layer and an output layer. The input–output relationship of this RBFNN network can be described by:

2 $$Y_{i} = \mathop \sum \limits_{j = 1}^{{N_{h} }} W_{ij} \varphi_{j} \left( x \right) + b_{i}$$

where $\varphi$ denotes the radial basis function of the hidden unit *j*; $x$ is the vector of input data; $W_{ij}$ is a weighted connections between output layer and the radial basis function; and $N_{h}$ represents the number of hidden-layer neurons. The constant term bi in Eq. (2) denotes a bias. As the activation function, an RBFNN hidden neuron has a Gaussian function.

3 $$\varphi_{i} \left( x \right) = exp\left( {{{ - x - c_{i}^{2} } \mathord{\left/ {\vphantom {{ - x - c_{i}^{2} } {2\sigma_{i}^{2} }}} \right. \kern-0pt} {2\sigma_{i}^{2} }}} \right), \quad i = 1, 2, N_{h}$$

where ${\text{c}}_{\text{i}}$ and $\sigma_{\text{i}}$ represent centers and widths, respectively, and $\left\| . \right\|$ denotes the Euclidean distance norm. The variances and centers are predefined and fixed for simplicity. From a point of view of design, the RBFNN networks training consists of finding the number of hidden layer nodes (neurons) Nh and the appropriate parameter set ($\sigma_{\text{i}}$, ${\text{W}}_{\text{ij}}$ and ${\text{c}}_{\text{i}}$) to map a given vector of input to a desired scalar of output efficiently with suitable accuracy and generalization. The supervised gradient-descent-based method [88, 89] was used for the network training.

#### Gene expression programming

GP is also utilized to model the behavior of structural engineering problems. It is a genetic algorithm extension which uses a program space for search, instead of employing a data space. One of the most important advantages of using GP-based methods over other approaches is their ability for production of prediction equations without any assumption for prior form of the relationship. GP as well as its variants have been employed by many researchers to discover any complex relationships which fit various experimental data [24, 25, 42] Researchers have introduced GEP as an efficient alternative approach to the traditional GP [45, 90]. Several different computer programs have been developed by GEP, through getting encoded in fixed length linear chromosomes, each of which composed of multiple encoding genes [45, 91].

GEP is rooted from evolutionary algorithms like GP and GA. A population of individuals is used in this method and then, better individuals are selected by employing genetic variations and fitness function. Genetic operators introduce the genetic variations. GEP as a learning machine, is supposed to learn the relationship between variables in groups of data. The difference between GEP and its precursors GP and GA is in the way of individual programming as in GEP, individuals are programmed as fixed length linear strings (chromosomes) which are shown eventually by expression trees which are a simple diagram presentation. However, individuals are expressed in GP and GA as fixed length linear strings (chromosomes) and nonlinear entities of different shapes (parse trees) and sizes, respectively. Genetic operators work at the chromosome level in GEP making creation of genetic diversity very simple, which is considered as one of its strengths over GP and GA. The unique, multi-genic nature of GEP is its other significant point allowing more complex programs with several sub-programs to be evolved. The benefits of both GP and GA are combined in GEP, while some of their limitations are met [39, 45, 92].

##### Architecture of GEP

**Fig. 2:**
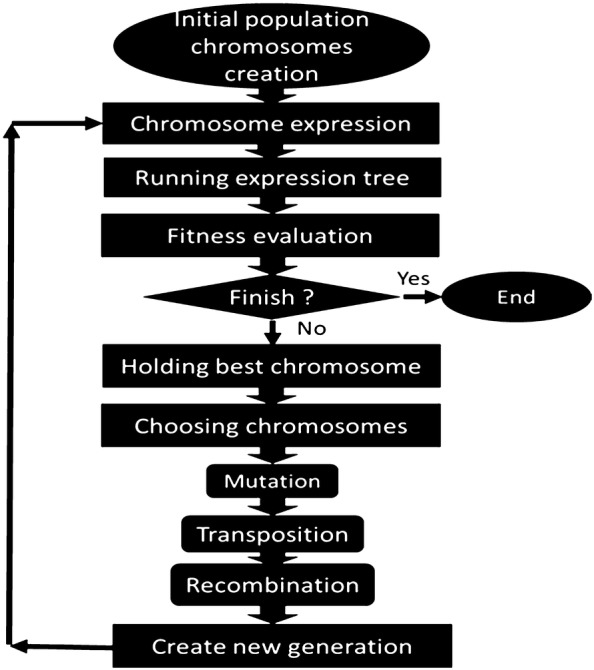
Gene expression programming diagram

In GEP the individuals are encoded as fixed size linear strings (chromosome), which are then shown as non-linear entities with different shapes and sizes, known as Expression Trees. For example, the expression of Eq. (4) can also be shown by an expression tree or diagram as represented in Fig. 2.

4 $$\left( {{\text{a }} + {\text{ b}}} \right) \, * \, \left( {{\text{c }} - {\text{ d}}} \right)$$

The actual phenotype of GEP chromosome is this representation and the genotype can be easily explained from the phenotype as shown in Eq. (4)

5 $$\begin{aligned} 0\; 1\; 2\; 3 \; 4 \; 5\; 6\hfill \\ *\; + \; - \;{\text{a}}\;{\text{b}}\;{\text{c}}\;{\text{d}} \hfill \\ \end{aligned}$$

As we read a page of text, the genotype is characterized as the straightforward reading of the expression tree from left to right and from top to bottom. The Eq. (5), for example, is an open reading frame which starts at ‘‘*’’ or position 0 and terminates at ‘‘d’’ or position 6. Karva notation or K-expression is the term describing these open reading frames [25, 93].

The GEP functional steps are shown in Fig. 2 [45]. The structure of GEP consists of function set, fitness function, terminal set, termination condition, and control parameters. As it can be seen in Fig. 2, an initial population is the start point of GEP. Then, the genes located on the chromosomes are expressed, and the fitness of each individual is analyzed. Subsequently, based on their fitness, the individuals are designated in order to reproduce with modification. The same process of development is performed on this new generation of individuals: the genomes expression, confrontation of the selection environment, and finally reproduction with modification. Altogether, this procedure is repeated for an exact number of generations or it is done until a termination condition is reached.

GEP system uses roulette wheel sampling with elitism to select and copy the individuals into the next generation based on the fitness. This guarantees that the best individuals are survived and cloned to the next generation. When single or several genetic operators are conducted on selected chromosomes, including rotation, mutation and cross over, variation is introduced in the population.

##### GEP methodology

The software package of GeneXpro was employed to run the GEP models. All parameters used in the GEP models are shown in Table 9.

**Table 9:** Parameters of GEP model

Parameter	Description of parameter	Setting of parameter
P1	Function set	$+ , - , \times , \div \surd ,3\surd ,sin,cos,Arctgx,$ x^2, x^3, e^x, *ln*, Inverse, Tanh, Avg 2 inputs
P2	Chromosomes	50
P3	Head size	8
P4	Number of genes	3
P5	Linking functions	Addition
P6	Fitness function error type	Root relative square error (RRSE)
P7	Mutation rate	0.044
P8	Inversion rate	0.1
P9	One-point recombination rate	0.1
P10	Two-point recombination rate	0.3
P11	Gene recombination rate	0.1
P12	Gene transportation rate	0.1

The mathematical operators and functions chosen in the present study are illustrative and not conclusive as the plant modeling designer as the freedom to choose such functions so as suit the anatomy of the problem under study. The operators and functions were chosen with a perspective of invoking simplicity of the evolved model promising faster convergence. The population size (number of chromosomes) sets the number of programs in the population. Larger population size takes longer for an iteration run. A large number of chromosomes were tested to find models with minimum error. The program was run until there was no significant improvement in the performance of the models. The present study was undertaken to attain explicit relationship between response variables and decision variables. GEP explicit formulations for PR, SL, STN, Vit and QI were obtained as a function of experimental parameters as follows:

PR, SL, STN, Vit and QI = f (KNO_3_, NH_4_NO_3_, Mesos, Micro, BA and IBA).

### Optimization of GEP models

#### Genetic algorithm model optimization

**Fig. 3:**
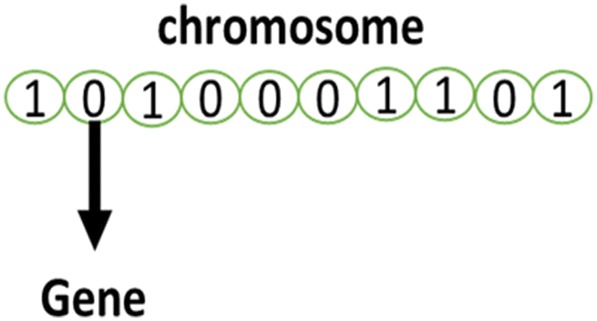
Structure of the chromosome and gene

GA is another search heuristic algorithm that is inspired by biological evolution in nature as well as Darwin’s evolution theory. This algorithm was first introduced by [94], and its development goes back to 1960–1970 [94, 95]. It is one of the oldest, most well-known, and most-widely used evolution algorithms utilized by researchers in different fields for the optimization of intricate problems. The algorithmic structure, like any of the other evolution algorithms, is composed of a population, where each individual in it is considered to be a solution to the problem. An individual is called chromosome and is composed of different problem variables that act as genes in the algorithm [91] (Fig. 3).

**Fig. 4:**
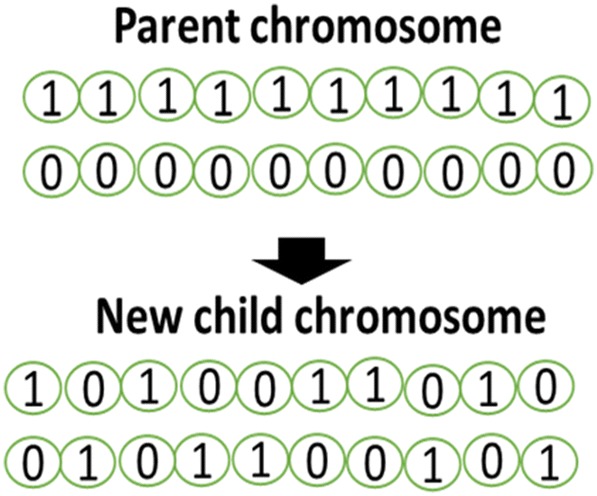
A crossover operator

The search procedure in this algorithm is created by developing a random population of chromosomes, and producing the next generation of the population is accomplished through three operators [91]:

Selection Operator: in this phase, the best chromosomes of the society, which are the best solutions to the problem, are identified by calculating the fitness function of each chromosome. The chromosomes are then used as parents for producing offspring, new child chromosomes and, thus, the next generation. Crossover Operator: using this operator, we produce offspring that are new child chromosomes from two parent chromosomes and take measurements to make this chromosome have better fitness than its parents. The crossover operator is in fact a method that determines the structure and ratio of the child’s chromosome compared with its parents’ chromosomes. The crossover operator can be implemented with different methods, including Ranking Selection, N-Point, Cycle, Order, Uniform, Tournament, and partially mapped [96]. Since use of a uniform method, such as the Crossover operator, is common between researchers [97] this method is utilized in the current research (Fig. 4).

**Fig. 5:**
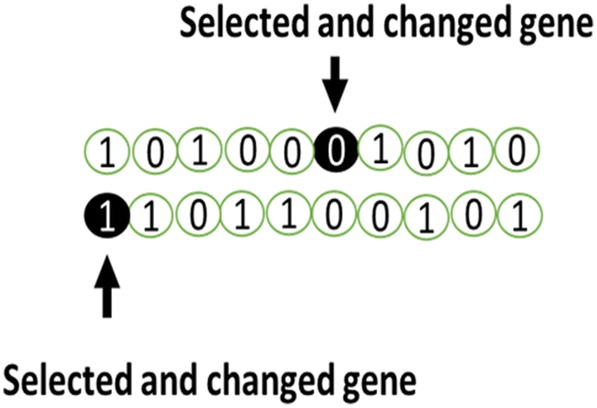
A mutation operator

Mutation Operator: this operator is utilized for searching new areas in the available dimension. The result makes the local optimum be not accepted as the best solution. To realize this goal, we need only to change some of the genes inside the chromosomes randomly (Fig. 5). Offspring new child chromosomes that are produced through these three operators are utilized as the next generation parents. The trend goes on as long as the termination condition determined by the researcher is met.

#### Statistical indices

In order to evaluate and compare the precision of different models, four statistical indices including root mean squared error (RMSE), mean absolute relative error (MARE), mean bias error (MBE) and correlation coefficient (R^2^) were used with the following formulas:

$$RMSE = \sqrt {\frac{{\mathop \sum \nolimits_{i = 1}^{N} \left( {O_{i} - P_{i} } \right)^{2} }}{N}}$$

$$MARE = \frac{1}{N}\mathop \sum \limits_{i = 1}^{N} \left| {\frac{{O_{i} - P_{i} }}{{O_{i} }}} \right|$$

$$MBE = \frac{1}{N}\mathop \sum \limits_{i = 1}^{N} \left( {O_{i} - P_{i} } \right)$$

$$R^{2} = \frac{{\mathop \sum \nolimits_{i = 1}^{N} \left( {O_{i} - \bar{O}_{i} } \right)\left( {P_{i} - \bar{P}_{i} } \right)}}{{\sqrt {\mathop \sum \nolimits_{i = 1}^{N} \left( {O_{i} - \bar{O}_{i} } \right)^{2} \mathop \sum \nolimits_{i = 1}^{N} \left( {P_{i} - \bar{P}_{i} } \right)^{2} } }}$$

where *O*_*i*_ and *P*_*i*_ are observed and predicted amounts, respectively; and $\bar{O}_{i}$ and $\bar{P}_{i}$ are mean observed and predicted amounts for N samples.

## Data Availability

The datasets used and/or analysed during the current study are available from the corresponding author on reasonable request.

## Abbreviations

MLPNN: multi-layer perceptron neural networks
MLR: multiple linear regression
RBFNN: radial basis function neural network
GEP: gene expression programming
GA: genetic algorithm
ANN: artificial neural networks
AI: artificial intelligence
GP: genetic programming
PR: proliferation rate
SL: shoot length
QI: quality index
STN: shoot tip necrosis
Vitri: vitrification
MS: Murashige and Skoog (1962)
BA: 6-benzylaminopurine
IBA: indole-3-butyric acid
